# Impact of Anti-Discoloration System (ADS) on the Optical Stability of Alkasite and Composite Resins: A Comparative Study on the Synergistic Staining Effect of Chlorhexidine and Coffee

**DOI:** 10.3390/ma19122506

**Published:** 2026-06-10

**Authors:** Tutku Baytok Kavcı, Münevver Söğüt Çetin, Hayal Boyacıoğlu, Lezize Şebnem Türkün, Murat Türkün

**Affiliations:** 1 Department of Restorative Dentistry, Faculty of Dentistry, Ege University, 35100 Izmir, Türkiye; mnvvrsgt@gmail.com (M.S.Ç.); sebnemturkun@gmail.com (L.Ş.T.); murat.turkun@ege.edu.tr (M.T.); 2 Department of Statistics, Faculty of Science, Ege University, 35100 Izmir, Türkiye; hayalboyacioglu@gmail.com

**Keywords:** color stability, dental polymers, chlorhexidine, ADS technology, CIEDE2000, alkasite, composite resins, coffee staining, extrinsic pigmentation

## Abstract

This study investigated the impact of conventional chlorhexidine (CHX) and anti-discoloration system (ADS)-containing CHX mouthrinses on the color stability of diverse dental polymers, both alone and in combination with coffee. Specimens (*n* = 180) were prepared from a nanohybrid composite (Charisma Diamond; Kulzer GmbH, Hanau, Germany), a monochromatic composite (Vittra Unique APS; FGM Dental Group, Joinville, Brazil), and a dual-cure alkasite (Cention Forte; Ivoclar Vivadent, Schaan, Liechtenstein). Following a 14-day cycle of mouthrinse immersion (2 min/daily) and coffee exposure (15 min/daily at 85 °C), color changes (ΔE_00_) were analyzed using the CIEDE2000 system. All materials exhibited significant discoloration across all protocols (*p* < 0.001). Cention Forte showed the highest susceptibility to staining, particularly in the CHX + coffee group (ΔE_00_ = 21.10), while Charisma Diamond remained the most stable (0.95–8.60). Conventional CHX (Kloroben; Drogsan Pharmaceuticals, Ankara, Turkey) induced significantly higher staining than ADS-CHX (Curasept ADS; Curasept S.p.A., Saronno, Italy) across all materials (*p* < 0.05). Notably, ADS technology significantly inhibited coffee-induced pigmentation in Cention Forte (*p* = 0.003) and Charisma Diamond (*p* = 0.046), effectively reducing the synergistic staining layer. In conclusion, while coffee consumption dramatically increases discoloration following CHX use, ADS technology serves as a protective barrier, reducing pigment adhesion. For patients with high dietary pigment intake, ADS-containing mouthrinses offer a significant clinical advantage in preserving the aesthetic longevity of polymeric restorations.

## 1. Introduction

In modern restorative dentistry, polymer-based composite resins are widely preferred due to their improved physical properties and optical characteristics like dental tissues [1]. However, the long-term success of these materials in the oral environment is directly dependent on the stability of the polymer matrix and the filler structure [2]. Water sorption and chemical degradation of polymeric chains impair the optical properties of the restoration, leading to color change (ΔE_00_), which is one of the primary causes of clinical failure [3].

Beyond its gold-standard role in periodontal therapy, chlorhexidine (CHX) is also a critical agent in preventive dentistry, frequently utilized in patients with high caries risk to significantly suppress Streptococcus mutans levels and reduce the overall bacterial load [4]. CHX gluconate exhibits high affinity for dental and polymer surfaces because it is a cationic molecule [5]. The adhesion of CHX to the surface prepares a ground for complex formation with dietary polyphenols and tannins (especially from beverages such as coffee and tea), resulting in severe extrinsic staining [6]. It has been reported in the literature that pigmented beverages consumed following CHX exposure create a “synergistic staining layer” on the polymer surface [7].

To prevent this aesthetic side effect, anti-discoloration system (ADS) technology aims to suppress oxidation and browning reactions through the synergistic combination of ascorbic acid and sodium metabisulfite, maintaining the anti-plaque efficacy of CHX while reducing extrinsic pigmentation [8]. Although the success of ADS technology on natural dental tissues has been well-documented, its protective efficacy on dental materials with different polymer architectures (monochromatic nanohybrids, low-shrinkage TCD-urethane systems, or ion-releasing alkasites) has not yet been fully elucidated [9].

Charisma Diamond stands out with its high molecular weight TCD-urethane monomer structure that provides low polymerization shrinkage, while Vittra Unique APS possesses a special filler technology that provides a monochromatic chameleon effect [10]. On the other hand, Cention Forte, a newly introduced ‘dual cure alkasite’ material, differs significantly from conventional polymer composite resins through its functional bioactivity [11]. While traditional composite resins prioritize optical stability and aesthetic longevity through highly cross-linked hydrophobic resin matrices and dense nano-hybrid filler configurations, alkasites are engineered to provide proactive therapeutic benefits via the continuous release of calcium, fluoride, and hydroxyl ions under acidic oral challenges [11]. However, this therapeutic ion-exchange mechanism requires a highly specific multi-monomer network integrated with large, reactive calcium fluorosilicate glass particles. The incorporation of hydrophilic monomers like PEG-400 DMA and a broader particle size distribution can inherently accelerate water sorption and hydrolytic degradation at the matrix–filler interface, creating potential trade-offs regarding its aesthetic longevity and resistance to extrinsic staining compared to conventional resin formulations. Evaluating the behavior of these distinct material architectures against CHX formulations with and without ADS under a heavy coffee consumption scenario is therefore of critical importance to guide clinical material selection based on both therapeutic and aesthetic durability.

The aim of this study is to comparatively evaluate the color stabilities of dental polymers with different monomer and filler structures under CHX mouthrinses (with and without ADS technology), either alone or in combination with coffee. The null hypotheses of the study are as follows: (I) there is no difference in discoloration among the polymeric materials used; (II) CHX formulations (ADS and conventional) show similar staining effects; (III) coffee consumption following mouthrinse use does not affect the degree of discoloration.

## 2. Materials and Methods

### 2.1. Material Selection

In this study, three different types of dental materials with distinct polymeric network structures and functional filler technologies were selected: their detailed compositions and technical profiles are summarized in Table 1.

The restorative materials investigated in this study were categorized into three main experimental groups based on their distinctive chemical formulations and structural categories, as summarized in Table 1. This classification was established to evaluate how different polymer structures—ranging from a conventional nanohybrid resin composite to a monochromatic sub-micron system and a bioactive alkasite material—influence the ultimate optical properties and color stability. The specific allocation and baseline characteristics of these main groups are detailed below:

**Group 1: Nano-hybrid composite resin (Charisma Diamond, Kulzer, Hanau, Germany)**
  ○ Polymer matrix: A bis-GMA-free organic matrix containing high molecular weight TCD-DI-HEA (tricyclodecane-urethane acrylate) and UDMA (urethane dimethacrylate). This specialized TCD matrix is designed to reduce polymerization shrinkage and increase cross-linking density.
  ○ Filler system: A nano-hybrid system consisting of barium aluminum fluorosilicate glass and fumed silica.
  ○ Filler content: High inorganic loading of 82% by weight (wt%) and 68% by volume (vol%).
  ○ Particle size: The particle size distribution ranges from 0.005 µm to 5 µm, with an average particle size of approximately 0.6 µm.
**Group 2: Monochromatic Composite Resin (Vittra Unique APS, FGM, Joinville, Brazil)**
  ○ Polymer matrix: A Bis-GMA-free resin system composed of UDMA, TEGDMA, and Bis-EMA. This matrix is formulated to provide high translucency to facilitate the “Chameleon Effect” (biomimetic color matching).
  ○ Filler system: Primarily consists of spherical zirconium silicate particles and nano-structured silica that optimize light scattering properties.
  ○ Filler content: Approximately 72% by weight (wt%) and 52% by volume (vol%) inorganic filler content.
  ○ Particle size: Contains sub-micron spherical fillers with an average particle size of 200 nm (0.2 µm), providing superior surface smoothness and optical mimicry.
**Group 3: Functional Bioactive Alkasite (Cention Forte, Ivoclar Vivadent, Schaan, Liechtenstein)**
  ○ Polymer matrix: A multi-monomer system including UDMA, DCP (tricyclodecane-dimethanol dimethacrylate), aromatic aliphatic UDMA, and PEG-400 DMA. This combination ensures high flexural strength and facilitates ion exchange.
  ○ Filler system: A complex bioactive filler blend containing calcium barium aluminum fluorosilicate glass, ytterbium trifluoride, and specialized “isofillers” (pre-polymerized composite fillers).
  ○ Filler content: Total inorganic filler loading is 78.4% by weight (wt%) and 57.6% by volume (vol%).
  ○ Particle size: Features a wide distribution range (0.1 µm to 35 µm); the presence of large isofillers supports structural integrity in bulk-fill applications.

The sample size for the study was determined via a priori power analysis using G*Power software (v3.1.9.7; Heinrich Heine University, Düsseldorf, Germany). Based on similar dental discoloration studies and literature data, the calculation utilized an effect size (f) of 0.40, a significance level (α) of 0.05, and a test power (1 − β) of 80%. According to the two-way ANOVA model, a total of 105 specimens (*n* = 7 per subgroup) was found to be sufficient. However, to increase statistical sensitivity, compensate for potential specimen loss, and more clearly capture minor differences between groups, the number of specimens in each subgroup was increased to *n* = 12 (total *n* = 180). A post hoc power analysis conducted at the end of the study confirmed that the findings possessed a statistical power exceeding 99%.

### 2.2. Specimen Preparation

A total of 180 disc-shaped specimens (*n* = 60/material) were prepared. For the immersion protocols, specimens from each main material group were randomly and equally divided into five distinct experimental subgroups (*n* = 12/subgroup), resulting in a total of 15 experimental subgroups across the entire study design. To ensure standardization, all preparation processes followed the protocol below:

- Standardization and Molding: Specimens were prepared using custom Teflon molds with apertures of 6 mm in diameter and 2 mm in height to simulate clinical restoration thicknesses. Composite resins were placed in bulk as a single increment. To prevent the formation of an “oxygen inhibition layer” and maximize surface smoothness, polyester strips (Mylar strip; SS White Co., Philadelphia, PA, USA) were placed on the top and bottom surfaces of the molds. Glass slides were then laminated over the strips and flattened under constant pressure to remove excess material. The Cention Forte capsule was then mixed for 15 s in a capsule mixer and placed in bulk into the prepared cavity using the manufacturer’s delivery gun. Light curing was performed for 20 s with an LED unit (1300 mW/cm^2^; ZenoLite, President, Allershausen, Germany).
- Polymerization Protocol: Photo-polymerization was performed using a high-intensity LED curing unit (1300 mW/cm^2^; ZenoLite, President, Allershausens, Germany) operating in a constant intensity mode. The tip of the light guide was positioned perpendicular to and in close contact with the specimen surface, and each side (top and bottom) was irradiated for 20 s according to the manufacturers’ instructions. To guarantee output consistency and avoid potential polymer under-curation, the light intensity was verified after every 10 specimens using a calibrated digital radiometer (Bluephase Meter II, Ivoclar Vivadent, Schaan, Liechtenstein).
- Surface Finishing and Polishing: Following polymerization, specimens were removed from the molds. To eliminate surface variations and achieve clinical standardization, a multi-stage finishing and polishing protocol was executed by a single operator. The FGM Diamond Pro (FGM, Joinville, Brazil) flexible aluminum oxide polishing disc system was applied sequentially using specific grit sizes: coarse (100 µm), medium (40 µm), fine (20 µm), and ultra-fine (2 µm). The discs were utilized with 10 parallel repetitions in the same direction at each stage under continuous water irrigation to prevent localized thermal degradation of the resin matrix. To prevent cross-contamination of abrasive particles between sequential grits, specimens were cleaned in an ultrasonic cleaner (Whaledent BioSonic, Coltene, Altstätten, Switzerland) containing distilled water for 30 s between discs. Final high-luster surface smoothening was achieved using the two-step, composite-specific Twist Dia (Kuraray Noritake, Okayama, Japan) diamond-impregnated flexible polishing wheels, utilizing the dark blue pre-polishing wheel followed by the light blue high-shine polishing wheel. Both wheels were operated under strict dry conditions without water spray for 30 s each at a standardized speed, adhering strictly to the manufacturers’ clinical instructions to optimize surface gloss and seal microscopic porosities.
- Pre-conditioning (Imbibition): To allow for complete water sorption, stabilization of the polymeric network, and balancing of post-polymerization reactions, all specimens were stored in an incubator (Memmert, Schwabach, Germany) in distilled water at 37 °C for 24 h prior to the initial (T0) color measurements.

### 2.3. Staining Protocol and Chemical/Chromogenic Cycling

A total of 180 specimens (*n* = 180) were prepared and initially divided into three main groups based on the restorative material used (*n* = 60 per material: Charisma Diamond, Vittra Unique APS, and Cention Forte). Within each material group, specimens were further randomly allocated into five experimental subgroups (*n* = 12/subgroup) to be subjected to a 14-day standardized chemical/chromogenic exposure cycle. These five subgroups consisted of: a staining control group (coffee), a conventional CHX group (Kloroben), an ADS-CHX group (Curasept ADS), a conventional CHX + coffee synergistic group, and an ADS-CHX + coffee synergistic group. All specimens across all subgroups were stored continuously in artificial saliva at 37 °C during the non-exposure periods of the daily cycles. This 3 × 5 × 12 factorial designs ensured a robust sample size for each interaction, providing a high statistical power (0.99) to detect significant differences in color stability (ΔE_00_).

Chromogenic Solution Preparation: Coffee solution was freshly prepared by mixing 2 g of instant coffee (Nescafe Classic, Nestlé, Vevey, Switzerland) with 200 mL of boiling distilled water. Immersion was performed at 85 °C to evaluate chemical sensitivity and pigment diffusion alongside the thermal expansion of the polymeric network. Specimens were immersed for 15 min daily.

Mouthrinse Applications: Mouthrinses used in the study were Kloroben (Drogsan Pharmaceuticals, Ankara, Turkey), containing conventional 0.12% chlorhexidine digluconate and 0.15% benzydamine HCl, and Curasept ADS 212 (Curasept S.p.A., Saronno, Italy), containing 0.12% chlorhexidine digluconate integrated with the anti-discoloration system (ADS). To facilitate a clear distinction for the reader, conventional chlorhexidine rinses lack anti-staining agents and promote pigment precipitation via non-enzymatic browning cascades, whereas the ADS technology chemically comprises a synergistic combination of ascorbic acid (acting as a competitive antioxidant) and sodium metabisulfite (acting as a powerful reducing agent) designed to actively intercept and block staining pathways directly on the material surfaces. Mouthrinses were placed in multi-well plates, and fresh solutions were utilized for each individual session.

Experimental Groups and Protocol:

1. Staining Control (Coffee): 15-min immersion in 85 °C coffee daily.
2. Kloroben Group: 2-min immersion daily.
3. Kloroben + Coffee Group: Specimens were first immersed in Kloroben for 2 min, rinsed with distilled water, and immediately transferred to 85 °C coffee for 15 min to simulate “chlorhexidine sensitization”.
4. Curasept ADS Group: 2-min immersion daily.
5. Curasept ADS + Coffee Group: Specimens were first immersed in Curasept ADS for 2 min, followed by a 15-min immersion in 85 °C coffee to test the protective effect of ADS technology.

Simulated Oral Environment (Artificial Saliva): Between immersion sessions, specimens were stored in artificial saliva at 37 °C in an incubator. The artificial saliva (pH 6.7) was formulated with NaCl (0.40 g/L), KCl (0.40 g/L), CaCl_2_·2H_2_O (0.79 g/L), NaH_2_PO_4_·2H_2_O (0.78 g/L), Na2S·9H_2_O (0.005 g/L), and urea (1.00 g/L). The solution was refreshed every 24 h [12].

### 2.4. Color Measurement and Spectrophotometric Analysis

Color measurements were performed at the baseline (T0) and after the 14-day cycle (T1) using a digital spectrophotometer (VITA Easyshade V; VITA Zahnfabrik, Bad Säckingen, Germany).

Color Measurement and Calibration: Color coordinates of all specimens were recorded at baseline and after the respective immersion cycles using a digital dental spectrophotometer (VITA Easyshade V, VITA Zahnfabrik, Bad Säckingen, Germany) To ensure high intra-examiner reliability and eliminate device drift, the spectrophotometer was strictly calibrated using its proprietary white calibration tile before each measurement session and automatically after every 10 specimens. All assessments were conducted under standardized D65 artificial interior lighting conditions by a single trained operator. Rather than utilizing the clinical shade-matching outputs, the device recorded the exact numerical Commission Internationale de l’Éclairage (CIE) L*, a*, and b* color coordinates driven by its internal narrow-band spectrophotometric sensor. To maintain consistency, measurements were taken at the geometric center of each specimen with the probe tip placed strictly perpendicular to the surface, ensuring full optical contact without applying excessive pressure. Specimens were positioned flat on a non-reflective standard white background to prevent any edge loss effect, ambient light scattering, or back-reflection confounding variations from the underlying surfaces. Three consecutive measurements were recorded for each specimen, and the average values of the CIE L*, a*, and b* coordinates were calculated. Total color change (ΔE_00_) was calculated using the CIEDE2000 formula, the current gold standard in the literature for its superior correlation with human visual perception [13].

### 2.5. Statistical Analysis

Descriptive and inferential data were analyzed using SPSS v25.0 (IBM Corp, Armonk, NY, USA). Depending on the normality of distribution, inter-group analyses were conducted using paired samples *t*-test and ANOVA, followed by Tukey and Tamhane post hoc tests. Significance was set at *p* < 0.05.

## 3. Results

### Descriptive Statistics and General Findings

Descriptive statistical analysis revealed that the type of material played a decisive role in staining susceptibility. Across all staining procedures, the highest ΔE_00_ values were observed in the Cention Forte group (ΔE_00_: 3.14–21.10), while Charisma Diamond exhibited the most stable optical performance (ΔE_00_: 0.95–8.60). However, when exposed solely to chlorhexidine (Kloroben), the highest discoloration was detected in the Vittra Unique APS (ΔE_00_: 5.24) material group (Table 2).

**Figure 1:**
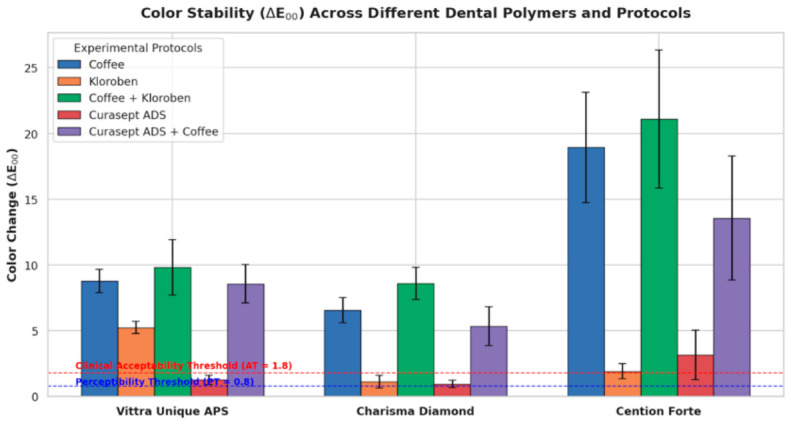
Total color change (ΔE_00_) values across different dental polymer materials subjected to standalone and synergistic chromogenic cycling protocols. The dashed blue line indicates the perceptibility threshold (PT = 0.8), and the dashed red line marks the clinical acceptability threshold (AT = 1.8). Error bars represent standard deviations.

In the CIEDE2000 (ΔE_00_) system, the perceptibility threshold (PT) is accepted as 0.8 units, while the clinical acceptability threshold (AT) is 1.8 units [14]. Comparing chlorhexidine formulations, Curasept, which incorporates anti-discoloration system (ADS) technology, demonstrated superiority in inhibiting pigmentation across all polymeric structures compared to conventional Kloroben. Specifically, in the Cention Forte polymer, Kloroben exposure resulted in a change of ΔE_00_: 21.10 units, whereas Curasept use reduced this change to ΔE_00_: 13.56, nearing clinical acceptability thresholds. Furthermore, the sequential use of chlorhexidine and coffee (Coffee + Kloroben) led to higher ΔE_00_ values in all materials compared to coffee use alone, confirming the formation of a chlorhexidine-induced “extrinsic staining layer” on the polymer surface (Table 2 and Figure 1).

Multiple comparison analyses (post hoc Tukey/Tamhane) showed that Curasept ADS provided the lowest color change across all polymeric structures and exhibited significantly more stable optical performance (*p* < 0.001) compared to conventional chlorhexidine (Kloroben). In inter-material evaluations, Cention Forte differed negatively from other materials across all solutions, while Vittra Unique APS and Charisma Diamond exhibited similar values, particularly under Kloroben and coffee exposure (*p* > 0.05).

One-way ANOVA among groups treated only with Kloroben mouthrinse showed highly significant differences in color stability between materials (F = 221.300; *p* < 0.001). According to Tukey post hoc analysis, all material groups differed significantly (*p* < 0.05). The highest color change was observed in the Vittra Unique APS (ΔE_00_: 5.24 ± 0.46) group, followed by Cention Forte (ΔE_00_: 1.91 ± 0.58) and Charisma Diamond (ΔE_00_: 1.12 ± 0.47).

In groups treated only with Curasept ADS mouthrinse, the difference in color stability between materials was significant (F = 13.565; *p* < 0.001). According to Tamhane post hoc analysis, no significant difference was found between the Vittra Unique APS (ΔE_00_: 1.25 ± 0.34) and Charisma Diamond (ΔE_00_: 0.95 ± 0.28) materials (*p* = 0.086). Conversely, Cention Forte (ΔE_00_: 3.14 ± 1.88) exhibited significantly higher color change than both materials (*p* < 0.05).

In groups exposed to the combination of Kloroben and coffee, the difference in color stability was significant (F = 51.026; *p* < 0.001). According to Tamhane post hoc analysis, no significant difference was detected between Vittra Unique APS (ΔE_00_: 9.81 ± 2.10) and Charisma Diamond (ΔE_00_: 8.60 ± 1.22) (*p* = 0.277). Meanwhile, Cention Forte (ΔE_00_: 21.10 ± 5.25) showed significantly higher color change compared to the other two materials (*p* < 0.001).

In groups exposed to the combination of Curasept ADS and coffee, the difference in color stability was significant (F = 23.284; *p* < 0.001). According to Tamhane post hoc analysis, all three material groups exhibited significantly different color change values (*p* < 0.05). The highest color change was observed in Cention Forte (ΔE_00_: 13.56 ± 4.71), followed by Vittra Unique APS (ΔE_00_: 8.57 ± 1.47) and Charisma Diamond (ΔE_00_: 5.33 ± 1.47).

Evaluation of chlorhexidine and coffee synergy: Differences between Kloroben and Kloroben + coffee exposures for each material group were analyzed using a paired samples *t*-test. The analysis revealed that in all groups, Vittra Unique APS (t = −7.806; *p* < 0.001), Cention Forte (t = −12.247; *p* < 0.001), and Charisma Diamond (t = −19.189; *p* < 0.001), the addition of coffee onto chlorhexidine mouthrinses significantly increased discoloration at a highly significant level. The most severe increase was observed in the Cention Forte group (Table 3).

Evaluation of ADS Technology and Coffee Interaction: Differences between Curasept ADS mouthrinse and Curasept + coffee combination were analyzed using the paired samples *t*-test. Findings showed that in Vittra Unique APS (t = −17.780; *p* < 0.001), Cention Forte (t = −7.391; *p* < 0.001), and Charisma Diamond (t = −9.995; *p* < 0.001), adding coffee to Curasept use significantly increased the color change values. However, these increases remained at lower levels compared to the conventional chlorhexidine (Kloroben) and coffee combination in Table 3.

Comparison of Coffee and Mouthrinse Combinations: When examining the differences between coffee exposure alone and coffee-mouthrinse combinations; no significant difference was found in the Vittra Unique APS (*p* = 0.195) and Cention Forte (*p* = 0.176) groups when Kloroben was added compared to coffee alone. In contrast, the Kloroben + coffee combination in the Charisma Diamond group exhibited significantly higher discoloration than coffee alone (*p* = 0.002; Table 3).

Protective Effect of ADS Technology on Coffee Staining: Comparing coffee exposure alone with the Curasept + coffee combination revealed that in the Cention Forte (*p* = 0.003) and Charisma Diamond (*p* = 0.046) groups, Curasept use significantly reduced color change compared to coffee exposure alone. In the Vittra Unique APS (*p* = 0.670) group, although a numerical decrease was observed, this difference was not statistically significant (Table 3).

## 4. Discussion

This study evaluated the optical stability of dental polymers with different chemical backgrounds against modern mouthrinse systems and aggressive staining agents using the CIEDE2000 formula. The findings revealed that both the material type and the mouthrinse-coffee combinations used created statistically significant differences in color change. Therefore, all null hypotheses (H01, H02, and H03) were rejected. In the CIEDE2000 (ΔE_00_) system, the perceptibility threshold (PT) is accepted as 0.8 units, while the clinical acceptability threshold (AT) is 1.8 units [14]. A critical finding of this study is that nearly all experimental groups exceeded the 0.8-unit PT, indicating that the color changes were clinically perceptible to the human eye. Furthermore, the 1.8-unit AT was significantly surpassed not only in the coffee-containing combinations, but also in most standalone mouthrinse groups. Specifically, Cention Forte exhibited the lowest optical stability, with ΔE_00_ values reaching up to 12 times the clinical threshold (21.10 units) when exposed to Kloroben and coffee. Even in the most stable interactions, such as Charisma Diamond and Vittra Unique APS with Curasept ADS, the values remained above the 0.8 PT, confirming that the chemical interaction between these polymers and mouthrinse systems poses a definitive risk to long-term aesthetic success.

All specimens in the study were kept in artificial saliva at 37 °C before the staining cycle and between sessions. Artificial saliva is critical for simulating the ionic balance and water sorption in the oral environment [15]. When polymeric matrices are exposed to artificial saliva, water molecules seep between the polymer chains, increasing the free volume and leading to plasticization of the matrix [16]. This process loosens the bonds between polymer chains, facilitating the diffusion of external pigments (such as coffee tannins) into the depths of the material. Especially in materials containing bioactive glass, such as Cention Forte, the ionic interaction and water sorption established with artificial saliva may have led to hydrolytic degradation at the filler–matrix interface, creating a surface area more susceptible to staining [17].

In this study, the highest color change in coffee exposure alone was observed in the Cention Forte (ΔE_00_: 18.93) group, while the lowest was in the Charisma Diamond (ΔE_00_: 6.55) group, both values exceeding the threshold limit. Coffee causes staining through both adsorption and absorption via tannins and polyphenols responsible for yellow discoloration [18].

In groups where mouthrinses were applied alone, statistically significant differences were found between conventional chlorhexidine (Kloroben) and Curasept containing ADS technology (*p* < 0.001). According to our findings, Kloroben created higher discoloration in all materials compared to Curasept. This behavior is governed by a multi-faceted molecular adsorption mechanism rather than an oversimplified electrostatic attraction. At experimental and physiological pH levels, conventional chlorhexidine operates as a highly dicationic biguanide molecule associated with two digluconate counter-ions. The initial baseline adherence is driven by long-range electrostatic forces between the protonated biguanide moieties of CHX and the polarized carbonyl oxygens present within the urethane or ester linkages of the polymer matrices. Concurrently, this stabilization is further reinforced by localized hydrogen bonding between the secondary amine networks (–NH–) of the CHX backbone and the resin matrix, as well as hydrophobic van der Waals interactions between the lipophilic outer 4-chlorophenyl rings of the biguanide and the hydrophobic aliphatic/aromatic chains of the dental monomers. Furthermore, as illustrated by the molecular structure of chlorhexidine digluconate, the presence of highly hydrophilic digluconate counter-ions containing multiple hydroxyl (–OH) groups enhances the local water affinity at the resin–solution interface. This structural feature potentially accelerates the water sorption-driven entrapment of dietary chromogens into the superficial layers of the polymer matrix, compounding the final staining severity [19].

Particularly, it is noteworthy that Vittra Unique APS exhibited the highest color change (ΔE_00_: 5.24) under Kloroben exposure alone, staining even more than Cention Forte. Vittra Unique APS contains a high rate of zirconium silicate fillers and a special UDMA/TEGDMA/Bis-EMA matrix structure to provide a monochromatic “chameleon” effect. In the literature, monomers such as TEGDMA are known to have high water sorption and hydrolytic sensitivity [20]. This specific matrix structure and zirconium content of Vittra Unique APS may have provided more binding sites for conventional CHX molecules to adhere. Conversely, the fact that ADS technology (Curasept) reduced the color change in the same material to a clinically acceptable level of ΔE_00_: 1.25 proves that the ascorbic acid and sodium metabisulfite in the ADS content successfully inhibited the oxidation and precipitation reactions of CHX on the polymer surface [21].

To fully elucidate the distinctive protective behavior of the ADS line against conventional chlorhexidine solutions like Kloroben, the underlying biochemical pathways must be characterized. Conventional biguanides, owing to their cationic nature, readily adsorb onto the resin matrix and expose free active sites that accelerate non-enzymatic browning cascades (Maillard reactions) when contacting dietary polyphenols and proteins [6]. The active components in Curasept ADS 212 mitigate this challenge through dual biochemical pathways. Sodium metabisulfite serves as a potent reducing agent that actively traps the intermediate reactive carbonyl compounds generated during the initial phases of the Maillard cascade, successfully halting the cross-linking required to synthesize insoluble, dark-colored melanoidin pigments. Concurrently, ascorbic acid functions as a competitive antioxidant that scavenges free radicals and blocks the oxidation pathways of complex dietary chromogens, such as coffee polyphenols. By intercepting these distinct chemical nodes, the ADS formulation structurally dampens pigment precipitation and subsequent chemical binding on the advanced polymer surfaces without altering the baseline antimicrobial properties of the core chlorhexidine molecule [21,22,23].

One of the most striking findings of this study is that coffee consumption following chlorhexidine (CHX) exposure increased color change at a highly significant level across all materials. In the literature, this is explained by CHX molecules adhering to the polymer surface and acting as a “magnet” for dietary chromogens [6]. The cationic structure of CHX forms ionic bonds with negatively charged polyphenols and tannins in coffee, creating an insoluble precipitation layer (extrinsic staining layer) on the surface [6].

The ΔE_00_ value of 21.10 detected in the Cention Forte group under the Kloroben + coffee combination was the highest discoloration level observed in our study. This extreme staining can be explained by the alkaline ion-releasing glasses in the structure of Cention Forte dissolving more under the acidic pH of coffee (approximately 4.5–5.0) and creating microscopic irregularities (porosities) on the surface [24]. In Cention Forte specimens kept in artificial saliva, these micro-voids formed during ion exchange may have caused coffee pigments modified with chlorhexidine to become trapped in the depths of the polymeric matrix.

Another surprising finding of our study is that ADS technology (Curasept) not only prevented its own staining but also significantly reduced the subsequent coffee staining. Specifically, in the Cention Forte material, the color change of ΔE_00_: 18.93 in coffee exposure alone decreased to ΔE_00_: 13.56 in the Curasept + coffee group. This suggests that the ascorbic acid and sodium metabisulfite present in the ADS content form a temporary film layer on the polymer surface, reducing the adhesion intensity of coffee pigments (anti-adhesion effect) [8,23].

A similar “protective effect” was observed in the Charisma Diamond group, where discoloration decreased from ΔE_00_: 6.55 to ΔE_00_: 5.33. The high surface density provided by the TCD-urethane structure of Charisma Diamond, combined with the protective barrier effect of ADS, minimized pigment penetration into the material [25]. These findings prove that for patients who consume extreme amount of coffee and must also use chlorhexidine mouthrinses, ADS technology offers a clinical advantage in preserving the aesthetic appearance of polymeric restorations. The color stability of restorative materials depends not only on their chemical structure, but also directly on their surface roughness (Ra) [26]. In this study, a multi-stage polishing protocol (FGM Diamond Pro and Twist Dia diamond-impregnated discs) was applied to all specimens to simulate clinical procedures. A smooth surface minimizes pits and protrusions where pigments can mechanically adhere [26]. The superior optical performance overall exhibited by Charisma Diamond across all groups can be explained by its nano-hybrid structure providing homogeneous wear and high polishability level [27]. On the other hand, the high discoloration values in the Cention Forte group suggest that the wide particle size distribution (up to 35 µm) may have left a more irregular morphology on the surface after polishing. Combined with ion release in artificial saliva, this may have reduced the surface’s resistance to staining of the material [28].

This study was conducted in a standardized laboratory environment and has some limitations. First, in vitro conditions may not fully reflect biofilm formation in the mouth, mechanical wear caused by chewing forces, and complex dynamic pH changes. However, to minimize these baseline limitations and maximize clinical relevance, artificial saliva was deliberately selected as the primary storage medium between immersion cycles instead of inert distilled water. Unlike water, artificial saliva contains essential inorganic ions and exhibits buffering capacities that closely dictate the hydrolytic degradation and superficial aging kinetics of resin matrices. This methodological rationale was particularly critical for accurately characterizing the bioactive alkasite material, whose ion-release mechanisms and dynamic surface topography are highly dependent on the surrounding ambient ionic concentration, thereby ensuring a more realistic intraoral simulation. Additionally, coffee consumption was simulated at an elevated temperature of 85 °C; this parameter was specifically selected to represent the maximum clinical serving peak of hot freshly brewed beverages and to evaluate dental polymers under an accelerated thermodynamic challenge. In polymer science, exposing cross-linked resin matrices to such temperatures induces kinetic chain relaxation and transient thermal expansion, which increases the free volume between polymer chains and lowers the energy barrier for pigment diffusion. This allows for an aggressive ‘worst-case scenario’ screening tool to characterize the materials’ ultimate chemical and structural resistance to combined thermal and chromogenic stress within a controlled laboratory setting. To contextualize these findings within a clinical perspective, the values must be compared against established optical thresholds. In contemporary dental literature utilizing the CIEDE2000 system, the clinical perceptibility threshold (PT) is defined as ΔE_00_ ≤ 0.8, while the clinical acceptability threshold (AT) is ΔE_00_ ≤ 1.8. In the present study, the highest discoloration value (ΔE_00_ = 13.56) vastly exceeded the clinical acceptability boundary (13.56 ≥ 1.8), signifying a severe aesthetic degradation that would be visually intolerable to patients and clinicians alike. Therefore, while such values demonstrate the ultimate staining susceptibility under an artificial ‘worst-case scenario’, they clinically forecast that synergistic chlorhexidine and hot coffee consumption can drastically shorten the aesthetic lifespan of these restorations, rendering them clinically unacceptable over time. However, this in vitro model constitutes a limitation as it may not fully replicate the dynamic intraoral clearing and temperature dissipation effect of natural saliva during physiological swallowing cycles. However, this aggressive scenario is valuable for observing the performance of dental materials under a worst-case scenario. Additionally, while specific guidelines such as ISO 22112 [29] exist for evaluating the discoloration of prefabricated artificial teeth, this study utilized established in vitro immersion protocols designed for direct resin-based restorative materials to investigate their behavior under simulated dietary and chemical exposure. Future studies are recommended to examine the long-term effects of ADS technology under different dietary habits and brushing cycles using in vivo or in situ models.

## 5. Conclusions

Within the limitations of this in vitro study, the following conclusions were reached:

1. **Material Factor**: Among all polymers tested, Charisma Diamond exhibited the highest color stability against both mouthrinses and coffee exposure. Cention Forte showed discoloration well above the clinical acceptability threshold (ΔE_00_ > 1.8), particularly in coffee combinations.
2. **Mouthrinse Effect**: Conventional chlorhexidine led to significantly more staining across all material surfaces compared to ADS technology.
3. **ADS Efficacy**: ADS technology not only prevented chlorhexidine-induced staining, but also significantly suppressed the adhesion of coffee pigments to the polymer surface.

## Figures and Tables

**Table 1:** Technical profiles and compositions of the restorative materials investigated.

Material	Manufacturer	Resin Matrix	Filler System	Particle Size (Reported by Manufacturer)	Filler Loading (wt%/vol%)	Lot No
**Charisma** **Diamond** (*Nanohybrid Resin Composite)*	Kulzer GmbH, Hanau, Germany	TCD-DI-HEA, UDMA.	Barium aluminum fluoride glass, colloidal silica.	0.005–1.5 µm *(Nanoparticles: 5–20 nm; Glass particles: 0.1–1.5 µm)*	82%/68%	10211
**Vittra Unique APS** (*Monochromatic Sub-micron Composite)*	FGM Dental Group, Joinville, Brazil	Bis-GMA, Bis-EMA, TEGDMA, UDMA.	Barium-aluminum silicate glass, silica nanoparticles.	Sub-micron spherical *(Average: 200 nm/0.2 µm)*	72%/52%	131223
**Cention Forte** (*Bioactive Bulk-fill Alkasite)*	Ivoclar Vivadent, Schaan, Liechtenstein	UDMA, DCP, tetramethylene glycol dimethacrylate, PEG-400 DMA.	Barium glass, ytterbium trifluoride, iso-fillers, calcium fluorosilicate glass.	0.1–35 µm *(Wide distribution bulk fillers)*	78.4%/57.7%	ZL12C4

Abbreviations: UDMA, urethane dimethacrylate; DCP, tricyclodecane-dimethanol dimethacrylate; PEG-400 DMA, polyethylene glycol-400 dimethacrylate; Bis-GMA, bisphenol A-glycidyl methacrylate; Bis-EMA, ethoxylated bisphenol A-dimethacrylate; TEGDMA, triethylene glycol dimethacrylate; TCD-DI-HEA, tricyclodecane-di-hydroxyethyl acrylate; wt%, weight percentage; vol%, volume percentage; APS, advanced polymerization system.

**Table 2:** Mean color change ΔE_00_ and standard deviation (SD) values of dental polymers subjected to different staining procedures.

Material Groups	Coffee (Mean ± SD)	Kloroben (Mean ± SD)	Coffee + Kloroben (Mean ± SD)	Curasept ADS (Mean ± SD)	Curasept ADS + Coffee (Mean ± SD)
**Vittra Unique APS**	8.79 ± 0.89 ^a^	5.24 ± 0.46 ^b^	9.81 ± 2.10 ^a^	1.25 ± 0.34 ^c^	8.57 ± 1.47 ^a^
**Charisma** **Diamond**	6.55 ± 0.95 ^a^	1.12 ± 0.47 ^b^	8.60 ± 1.22 ^a^	0.95 ± 0.28 ^b^	5.33 ± 1.47 ^c^
**Cention Forte**	18.93 ± 4.19 ^a^	1.91 ± 0.58 ^b^	21.10 ± 5.25 ^a^	3.14 ± 1.88 ^b^	13.56 ± 4.71 ^c^

Different superscript letters (a, b, c) within the same row indicate statistically significant differences between the experimental protocols for each specific restorative material (*p* < 0.05) according to the Tukey HSD post hoc test. Identical letters indicate no statistically significant difference (*p* > 0.05).

**Table 3:** ΔE_00_ analysis of differences created by mouthrinses compared to coffee exposure alone.

Material	Coffee vs. (Kloroben + Coffee) (ANOVA/Post Hoc)	Coffee vs. (Curasept + Coffee) (ANOVA/Post Hoc)	Curasept ADS Alone vs. (Curasept + Coffee) (Paired *t*-Test)
**Vittra Unique APS**	Non-significant (*p* = 0.195)	Non-significant (*p* = 0.670)	Significant Increase (t = −17.780; *p* < 0.001)
**Charisma Diamond**	Significant Increase (*p* = 0.002)	Significant Decrease (*p* = 0.046)	Significant Increase (t = −9.995; *p* < 0.001)
**Cention Forte**	Non-significant (*p* = 0.176)	Significant Decrease (*p* = 0.003)	Significant Increase (t = −7.391; *p* < 0.001)

## Data Availability

The original contributions presented in this study are included in the article. Further inquiries can be directed to the corresponding authors.
